# Supplementary material for: HIVprotI: an integrated web based platform for prediction and design of HIV proteins inhibitors
Source: J Cheminform. 2018 Mar 9;10:12. doi: 10.1186/s13321-018-0266-y (PMC5845081; doi:10.1186/s13321-018-0266-y)
Supplement: Supplementary file 2 — Additional file 2. Source code of HIVProtI web server. [file 13321_2018_266_MOESM2_ESM.zip › HIVprotI_Source-code/hivprotI/weka-3-6-6/doc/weka/core/neighboursearch/balltrees/package-summary.html]

weka.core.neighboursearch.balltrees


---


|  |  |  |
| --- | --- | --- |
| **Overview** \| **Package** \| Class \| **Tree** \| **Deprecated** \| **Index** \| **Help** \| **Weka's home** | |  |
| **PREV PACKAGE**   **NEXT PACKAGE** | **FRAMES**    **NO FRAMES**     **All Classes** |  |


---

## Package weka.core.neighboursearch.balltrees

| **Class Summary** | |
| --- | --- |
| **BallNode** | Class representing a node of a BallTree. |
| **BallSplitter** | Abstract class for splitting a ball tree's BallNode. |
| **BallTreeConstructor** | Abstract class for constructing a BallTree . |
| **BottomUpConstructor** | The class that constructs a ball tree bottom up. |
| **MedianDistanceFromArbitraryPoint** | Class that splits a BallNode of a ball tree using Uhlmann's described method.    For information see:    Jeffrey K. |
| **MedianOfWidestDimension** | Class that splits a BallNode of a ball tree based on the median value of the widest dimension of the points in the ball. |
| **MiddleOutConstructor** | The class that builds a BallTree middle out.    For more information see also:    Andrew W. |
| **PointsClosestToFurthestChildren** | Implements the Moore's method to split a node of a ball tree.    For more information please see section 2 of the 1st and 3.2.3 of the 2nd:    Andrew W. |
| **TopDownConstructor** | The class implementing the TopDown construction method of ball trees. |

---


|  |  |  |
| --- | --- | --- |
| **Overview** \| **Package** \| Class \| **Tree** \| **Deprecated** \| **Index** \| **Help** \| **Weka's home** | |  |
| **PREV PACKAGE**   **NEXT PACKAGE** | **FRAMES**    **NO FRAMES**     **All Classes** |  |


---
